# “I wanted to know what was hurting so much”: a qualitative study exploring patients’ expectations and experiences with primary care management

**DOI:** 10.1186/s12891-023-06885-x

**Published:** 2023-09-26

**Authors:** Véronique Lowry, François Desmeules, Diana Zidarov, Patrick Lavigne, Jean-Sébastien Roy, Audrey-Anne Cormier, Yannick Tousignant-Laflamme, Kadija Perreault, Marie-Claude Lefèbvre, Simon Décary, Anne Hudon

**Affiliations:** 1 School of Rehabilitation, Medicine Faculty, University of Montreal, 5415 L’ Assomption Boulevard, Pav. Rachel Tourigny, Montreal, Canada H1T 2M4; 2 Centre Intégré Universitaire de Santé Et de Services Sociaux (CIUSSS), de l’Est-de-L’Île-de-Montréal, Montréal, Canada; 3 Centre de Recherche Interdisciplinaire en Réadaptation (CRIR), Montreal, QC Canada; 4 Institut Universitaire Sur La Réadaptation en Déficience Physique de Montréal (IURDPM), Montréal, Québec Canada; 5 Department of Surgery, Faculty of Medicine, University of Montreal, Montreal, QC Canada; 6 Department of Rehabilitation, Faculty of Medicine, Université Laval, Québec City, Québec Canada; 7 Centre Interdisciplinaire de Recherche en Réadaptation Et Intégration Sociale (Cirris), Quebec City, QC Canada; 8 School of Rehabilitation, Medicine Faculty, University of Sherbrooke, Sherbrooke, Canada; 9 Centre de Recherche du CHUS, Université de Sherbrooke, Sherbrooke, Canada; 10 Groupe de Médecine Familiale Universitaire (GMF-U) Maisonneuve-Rosemont, Montréal, Canada; 11 Centre de Recherche en Éthique (CRÉ), Montréal, QC Canada

**Keywords:** Shoulder, Expectation, Experience, Recommendations, Clinical practice guidelines, Qualitative research

## Abstract

**Background:**

The management of shoulder pain is challenging for primary care clinicians considering that 40% of affected individuals remain symptomatic one year after initial consultation. Developing tailored knowledge mobilization interventions founded on evidence-based recommendations while also considering patients’ expectations could improve primary care for shoulder pain. The aim of this qualitative study is to explore patients’ expectations and experiences of their primary care consultation for shoulder pain.

**Methods:**

In this qualitative study, participants with shoulder pain and having consulted a primary care clinician in the past year were interviewed. All the semi-structured interviews were transcribed verbatim, and inductive thematic analysis was performed to identify themes related to the participants’ expectations and experiences of primary care consultations for shoulder pain.

**Results:**

Thirteen participants with shoulder pain were interviewed (8 women, 5 men; mean age 50 ± 12 years). Eleven of them initially consulted a family physician or an emergency physician, and two participants initially consulted a physiotherapist. Four overarching themes related to patients’ expectations and experiences were identified from our thematic analysis: 1) *I can’t sleep because of my shoulder*; 2) *I need to know what is happening with my shoulder*; 3) *But… we need to really see what is going on to help me!*; and 4) *Please take some time with me so I can understand what to d*o*!*. Several participants waited until they experienced a high level of shoulder pain before making an appointment since they were not confident about what their family physician could do to manage their condition. Although some participants felt that their physician took the time to listen to their concerns, many were dissatisfied with the limited assessment and education provided by the clinician.

**Conclusions:**

Implementing evidence-based recommendations while considering patients’ expectations is important as it may improve patients’ satisfaction with healthcare. Several participants reported that their expectations were not met, especially when it came to the explanations provided. One unexpected finding that emerged from this study was the waiting period between the onset of shoulder pain and when patients decided to consult their primary care clinician.

**Supplementary Information:**

The online version contains supplementary material available at 10.1186/s12891-023-06885-x.

## Background

Shoulder disorders are common, with a yearly incidence reported to be as high as 55% [1]. Shoulder pain is also one of the third most common musculoskeletal disorders in the working population and this condition results in important disability and loss of productivity [2]. Several individuals will present persistent shoulder pain, with 40% of adults that are still symptomatic one year after their initial consultation in primary care [3]. In this context, shoulder disorders should be managed timely, considering that patients consulting with persistent shoulder pain suffer from significantly higher levels of pain and disability and have a poorer quality of life compared to patients that were managed earlier in the acute phase [4]. An optimal management of shoulder pain in primary care is crucial and could reduce the risk of long-term disability [5, 6]. To improve the early management of shoulder pain, evidence-based recommendations from shoulder clinical practice guidelines (CPGs) need to be efficiently implemented by also considering contextual factors that may limit the applicability and uptake of recommendations [7].

Common shoulder pain diagnoses include rotator cuff-related shoulder pain (RCRSP), adhesive capsulitis, glenohumeral (GH) instability, GH osteoarthritis and acromioclavicular disorders. Recommendations for the management of most shoulder disorders include that shoulder pain should be diagnosed using a detailed clinical examination; combining a history of the injury and subjective assessment of symptoms, as well as physical examination [8, 9]. Despite some diagnostic uncertainties due to a lack of sensibility and specificity of physical examination tests [10, 11], recent evidence from CPGs indicates that diagnostic imaging is discouraged for the initial management of shoulder pain [12–14]. Diagnostic imaging tests should be reserved for shoulder pain cases in which there is a suspicion of a serious pathology requiring urgent or specialized care [12–14]. Moreover, a referral to a medical specialist such as an orthopedic surgeon is not indicated unless there are red flags or specific indications, such as the suspicion of an acute full-thickness traumatic rotator cuff tear in a young active patient [12–14]. Indeed, according to several high-quality CPGs, the majority of shoulder disorders can be treated in primary care with conservative management, including active rehabilitation and the short-term use of medication such as non-steroidal anti-inflammatory drugs (NSAIDs) or acetaminophen [13, 15, 16].

However, implementing recommendations from CPGs may be more challenging when patients’ expectations are not aligned with recommended management. For example, patients consulting in primary care, including patients with musculoskeletal disorders, often expect a prescription for diagnostic imaging or a referral to a medical specialist [17, 18]. When their expectations are unmet, patients living with shoulder pain often report anxiety and dissatisfaction with their care [19, 20]. However, prescribing tests or treatments that are not in agreement with evidence-based recommendations may lead to negative outcomes and increased healthcare costs [19, 21]. Patients’ expectations can affect primary care management as primary care clinicians have reported that they sometimes intentionally did not follow evidence-based CPGs recommendations because of patients’ demands [22, 23]. Primary care clinicians reported agreeing to patients’ demands to avoid hindering the therapeutic relationship or because there was not enough time to provide education [23]. These “guideline-incoherent” decisions (e.g., prescribing diagnostic imaging tests) have been shown to increase healthcare costs and the risks of unfavourable patients’ outcomes [24, 25]. Ultimately, we need to improve our understanding of the expectations of shoulder pain patients when they consult for primary care as it can affect outcomes [26].

Patients’ expectations and experiences of shoulder pain management have mostly been studied for specific shoulder conditions, such as adhesive capsulitis, or for a specific approach, such as exercise therapy [19]. To our knowledge, there is only one qualitative study on patients’ perspectives of primary care for shoulder pain and participants identified that receiving a diagnosis, discussing management options and prognosis as well as the need for reassurance were their priorities [27]. Moreover, there has been no study on patients’ opinions of shoulder primary care management within the Canadian healthcare system. Considering that patients’ expectations and experiences of healthcare services are associated with the specific context within which these services are received, exploring patients’ expectations and experiences in the Canadian setting is necessary to inform a tailored efficient implementation of recommendations from CPGs.

Therefore, the aim of this qualitative study is to explore the expectations and experiences of patients towards primary care consultations for their shoulder pain.

## Methods

### Study design and ethics

This qualitative study follows the Consolidated criteria for reporting qualitative research (COREQ) checklist [28]. This study is part of a larger project aiming to develop a knowledge mobilization intervention to facilitate the management of shoulder pain in primary care by implementing recommendations from high quality CPGs [13, 15, 29–33]. This larger study included focus groups with primary care clinicians (i.e., family physicians and physiotherapists) to explore barriers and facilitators to the implementation of recommendations. In the current project, to achieve a better understanding of patients’ perspectives and inform the design of the implementation intervention, we conducted semi-structured interviews exploring patients’ expectations and experiences when it came to the management of their shoulder pain. We conducted semi-structured interviews between August 2021 and December 2021 with patients who consulted a primary care clinician for shoulder pain during the previous year. The study was approved by the Health Research Ethics Committee of the *Centre intégré universitaire de santé et de services sociaux* (CIUSSS) de-l’Est-de-l’Île de Montréal (2021–2224) in Montreal, Quebec, Canada.

### Sampling and recruitment of participants

Patients were eligible to participate in the study if they: 1) were 18 years or older; 2) had consulted a primary care clinician for shoulder pain in the last year; 3) were able to communicate orally in French. Participants could still have shoulder pain or have recovered. There were no specific exclusion criteria. Convenience sampling was used for recruitment, using different strategies. Patients who saw their primary care physician during the previous year were identified by orthopedic surgeons from a large urban Montreal hospital outpatient clinic. We also sent an email to physicians practicing in University Family Medicine Groups (*n* = 3) as well as to physiotherapists from various private physiotherapy clinics (*n* = 59) in the province of Quebec, that have accepted to be contacted, to identify patients that could be included in the project. Moreover, we posted invitations for primary care clinicians to reach out to potential participants through social media accounts. Information explaining the project and the inclusion criteria was provided to clinicians with an email address to contact the research team. We used different techniques to include a wide array of patients with different shoulder disorders and a varied experience of primary care. Individuals wanting to take part in the study contacted the research team by email.

### Data collection

A semi-structured interview guide, including open-ended questions about patients’ expectations and experiences was developed by our team. The interview guide was adapted from the one used in an Irish study aiming to explore the views and experiences of patients living with shoulder pain [34]. The interview guide from this previous study was developed with the involvement of stakeholders; a healthcare practitioner and a patient living with shoulder pain [34]. The guide was adapted by members of the research team with over seven years of clinical experience in the treatment of shoulder pain patients (VL, FD) and in conducting qualitative research (DZ, KP, AH). Themes addressed in the semi-structured interview guide focused on patients’ history and the impacts of shoulder pain, the perceived cause of shoulder pain, the reasons why they decided to consult, their expectations about management and rehabilitation, as well as primary care consultation experience and input on shared decision-making. After two interviews, the guide was revised by the research team and only minor modifications were made to ensure the fluidity of the interviews. The interview guide is available as Supplementary Material. The interviews were conducted virtually using Zoom Meeting Education (Zoom, San Jose), a secure platform using end-to-end encryption, and were recorded via the Zoom platform. Interviews were also conducted over the phone, depending on the participant preference. There was a single interview with each participant. Two interviews were conducted over the phone and 11 interviews were conducted using the Zoom platform. Interviews were conducted by a physiotherapist and PhD candidate who identifies as a woman (VL). The interviewer had no previous experience in conducting interviews in the context of qualitative research but had seven years of experience in the clinical management of shoulder pain at the time of the interview. However, prior to and during the collection of data, the interviewer was mentored by three members of the research team with extensive experience in conducting interviews for qualitative studies (DZ, KP, AH).

For feasibility reasons and since a systematic review concluded that saturation was generally achieved after nine to 17 individual interviews, we had planned to do between 10 and 14 interviews [35]. We stopped the interviews after meeting 13 participants, since no new concepts emerged from the interviews, according to the interviewer in charge of the study (VL). Two interviews were transcribed by one author (VL) and eleven interviews were transcribed using a professional transcription service. Transcripts were not returned to the participants.

### Data analysis

Transcripts were analyzed using inductive thematic analysis based on Braun and Clarke’s 6-step approach: 1) Reading and becoming familiar with data; 2) Generating initial codes; 3) Searching for themes; 4) Reviewing themes; 5) Defining and naming themes; 6) Producing the report [36]. We have used a constructivist epistemological approach for the study [37]. From a constructivist standpoint, it is acknowledged that the participants' perceptions and experiences shape the reality they describe, and knowledge is co-constructed during the interviews [37]. Two team members (VL, ACC) reviewed the transcripts to verify the accuracy of the transcription. Then, they both inductively coded two transcripts using NVivo 12 (QSR International Pty Ltd.) and compared and discussed their initial codes. AAC then proceeded to code all the other interviews. VL reviewed and modified the codes and generated a coding tree. Preliminary themes were identified by VL through the codes and were organized into broader themes and subthemes using a conceptual map. No a priori themes or conceptual frameworks were used, as themes were inductively derived from the data. The first author in charge of the analysis also consulted the senior author, an experienced qualitative researcher (AH) at different time points during the analysis to improve the organization and conceptualization of the themes. Relevant citations that were included in the manuscript were translated from French into English by a professional translator and verified by the first author (VL).

## Results

The thirteen interviews lasted a mean (standard deviation) of 38 min (± 8 min). Eight women and five men were interviewed, and the median age of participants was 48 years old (range: 34 to 68). Ten participants consulted a family physician, one participant consulted an emergency physician, and two participants consulted a physiotherapist when seeking primary care for their shoulder pain. Symptoms appeared following trauma in four participants while the onset was progressive in the nine others. Sociodemographic and clinical characteristics of the participants are presented in Table 1.

**Table 1:** Sociodemographic and clinical characteristics of participants (*n* = 13)

	n (%)^*^
**Age (years)**^**+**^	48 (34–68)
**Gender**	8 women (62) 5 men (38)
**Self-reported shoulder pain diagnosis**	
RC tendinopathy/Bursitis	3 (23)
RC tear	1 (8)
RC and LHB tear	2 (15)
GH osteoarthritis	1 (8)
GH instability	1 (8)
Adhesive capsulitis	2 (15)
Calcific tendinitis	2 (15)
Unknown	1 (8)
**Shoulder pain duration (months) **^**+**^	29 (2–84)
**Time since first consultation with a healthcare clinician (months) **^**+**^	12 (1–26)
**Time between onset of shoulder pain and first consultation with a healthcare clinician (months) **^**+**^	8 (0–66)
**First healthcare clinician consulted**	
Family physician	10 (77)
Emergency physician	1 (8)
Physiotherapist	2 15)
**Dominant shoulder affected**	11 (85)
**Marital status**	
Single	4 (31)
Married/Common law union	9 (69)
**Education level completed**	
High school	2 (15)
College	6 (46)
University	5 (38)
**Employment status**	
Employed	10 (77)
Pension/Part-time job	2 (15)
Pension	1 (8)
**Annual income**	
Less than 20 000$	1 (8)
20 000 – 30 000$	1 (8)
40 000 – 50 000$	5 (38)
60 000 – 70 000$	2 (15)
More than 70 000$	3 (23)
Unknown	1 (8)

*RC* Rotator cuff

*LHB* Long head of biceps

*GH* Glenohumeral

^*^Data are presented as the number of participants that met the characteristic unless otherwise mentioned. Percentages are in parentheses

^**+**^Data are presented as median (range)

### Themes identified

**Fig. 1:**
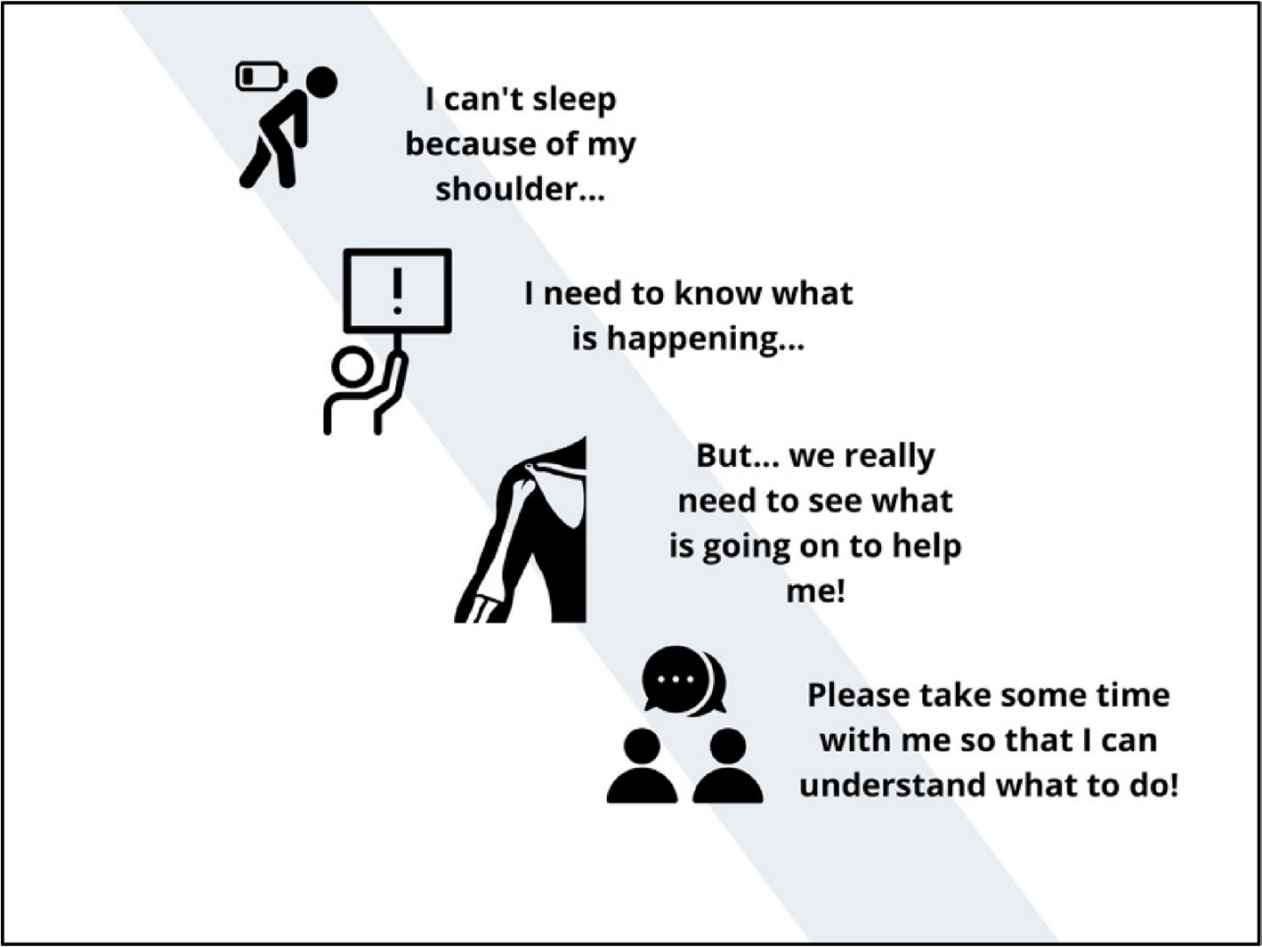
Main themes identified regarding participants’ expectations and experiences

Four overarching themes related to patients’ expectations and experiences were identified from our thematic analysis: 1) *I can’t sleep because of my shoulder*; 2) *I need to know what is happening with my shoulder;* 3) *But… we need to really see what is going on to help me!*; and 4) *Please take some time with me so I can understand what to do!*. These themes are presented in Fig. 1 and in the following section with quotes for a detailed presentation.

### Theme 1: *I can’t sleep because of my shoulder*

Most participants reported that they consulted a primary care clinician for their shoulder pain because they could not sleep, they noticed a change in their mood, there was an important disruption in their daily activities, or they were unable to work. Participants also reported that their pain was worsening. Participants waited a median of eight months after the onset of shoulder pain to consult their primary care clinician.

A participant reported that she was showing signs of irritability because she was not able to sleep:

“And that was also the reason why I decided to go see a physiotherapist. Because I realized that now, it had started to change my behaviour a bit and I really had a much shorter fuse. And that’s not the kind of person I am in life. […] So then, when you start showing signs of losing your patience in situations when normally you’re alright, you say to yourself: “Yes, maybe not sleeping at night is not helping me…”.” (PT03, woman, glenohumeral instability).

Another participant (PT05, woman, adhesive capsulitis) mentioned that she felt she was losing her independence and that she was unable to solve her shoulder problem by herself.

Various reasons were reported by participants to explain the delay between the onset of shoulder pain and their first consultation in primary care and that they waited until the pain was debilitating.

Many participants said they did not feel confident that their family physicians would be able to do anything to manage their shoulder pain, or thought that they would receive a prescription for a treatment that did not meet their expectations, as mentioned by two participants:

“I would say to myself: what is he going to do with that? What can he do with that? Take medication again…” (PT05, woman, adhesive capsulitis).

“Maybe… For me, I thought she would give me pain medication or just like anti-inflammatory drugs. […] maybe my doctor, […] she usually goes to medication right away. I do not really know why […]” (PT12, woman, RC tendinopathy/bursitis).

Some participants also believed that the lack of insurance to cover the costs of physiotherapy treatments was a barrier to seeking care for their shoulder pain, as mentioned by a participant:

“And my physiotherapy was not covered by my insurance. Which is also maybe why I waited before going for a consultation also.” (PT03, woman, glenohumeral instability).

In brief, participants waited until the pain became an important limitation, such as preventing them from sleeping or difficulties with the activities of daily living, prior to consultation because they thought the shoulder pain would resolve spontaneously. They did not feel confident about the options available with the primary care management of shoulder pain or did not have access to physiotherapy.

### Theme 2: *I need to know what is happening with my shoulder*

An important theme, identified throughout the analysis of participants’ transcripts, was that the participants needed to know what was wrong with their shoulder. Most participants identified this need as the reason to consult a healthcare clinician or what they expected their first consultation with a primary care clinician to provide.

A participant mentioned: “*I wanted to know what I had… What was hurting so much.*” (PT04, man, RC tear).

A participant directly stated that her main reason to consult a physiotherapist was to get a diagnosis:

*“[…] that was really the main reason. It was, one, to know what my physiotherapy diagnosis is. […] I think that […] the clinical opinion is important.”*

(PT03, woman, glenohumeral instability).

Other participants explicitly indicated expecting the clinician to perform a physical assessment and touch their shoulder to really understand what was wrong with it. (PT10, man, diagnosis unknown) mentioned:

*“…What did I expect? Well… well, probably that he… he would touch my shoulder, and maybe try to move it or to… stretch it, or I don’t know what. […] To try and see if something hadn’t shifted out of place […]”*

However, despite participants’ expectations to undergo a clinical examination and to receive a diagnosis for their shoulder pain, many of them felt that the clinician did not, or only minimally, assessed their shoulder.

A participant explained that her family physician did not touch her shoulder and perceived that the clinical exam performed was insufficient, leaving her with uncertainties regarding her shoulder diagnosis. She mentioned:

“And then COVID changed him, in a negative way. […] he never touched my arm. He really kept me at a distance. […] he really didn’t want us to come close and all that. So, he said to me: “Good, move like that. Move this other way. So, we can see that your movements are limited. So, it must be tendinitis” (PT05, woman, adhesive capsulitis).

Some participants stated that they did not receive a diagnosis from the primary care clinician for their shoulder pain and that they were referred for diagnostic imaging without a clinical examination or further explanations.

“Well, at first, he did not really make a diagnosis, he said: “Ah, we will go… we will look a bit further”. So he sent me… he sent me for X-rays and an ultrasound.” (PT08, woman, RC tendinopathy).

Another participant was unsatisfied with the lack of specificity in the diagnosis made by the family physician:

“No, even before, he did not tell me his diagnosis, it was after doing the cortisone injection that he said that maybe it was bursitis, tendinitis, he basically named them all. I would have liked to get a bit more then and get a more specific diagnosis for my injury […]me the average human being that doesn’t know medicine, well I would have liked to have something more concrete in his diagnosis.” (PT07, man, RC tendinopathy/bursitis).

Conversely, some participants reported a positive experience regarding the initial management of their shoulder pain in primary care. One participant mentioned that his family physician took the time to ask questions, perform a clinical exam and provide information regarding treatment.

“He took the time to ask me all kinds of questions. And we had almost 45 min together. […] So, he really took the time to, to look at how I was holding myself, how the shoulder was compared to the other. He examined all that, raised my arms… with resistance. So he really did some…some… a little examination and some… and some tests. He asked me the exact location of the pain, how incapacitating it was.” (PT11, woman, RC calcific tendinitis).

Overall, these citations highlight that participants expected their primary care clinician to perform a clinical examination to reach a diagnosis. Many participants were not satisfied with their clinical examination, reported that they did not receive a diagnosis or that they were not satisfied with the specificity of the diagnosis provided. Participants appreciated when their care provider asked them questions about their shoulder pain and performed a clinical examination.

### Theme 3: *But… we need to really see what is going on to help me!*

As previously mentioned, most participants wanted to know what was wrong with their shoulder and expected to receive a diagnosis. However, several participants believed that imaging tests, such as radiographs, were the only way to really see what the problem was and how to deal with it. Six participants expected their primary care clinician to refer them for diagnostic imaging.

Some participants clearly stated that they wanted a picture of their shoulder to be taken to understand what was causing their pain. One participant mentioned:

“Well because when we have something that is displaced, we expect that… to have an image of what’s inside of… inside of our body, basically.” (PT10, man, unknown diagnosis).

A second participant mentioned:

“Well for me, I would like it better if I could figure it out myself. I would rather have an image, you know, a picture. A picture of yes that’s it, look, that’s it. That, that would make me feel more secure and I would say OK, we are going somewhere, and we will fix it by doing this and doing that.” (PT07, man RC tendinopathy/bursitis).

The same participant was not confident that the physician could provide a diagnosis without X-rays because he could not see the shoulder’s structures:

“If, let’s say the doctor had come and taken an X-ray to say OK that’s it, […] what is going on underneath we can’t see it if […] we don’t take an X-ray or something.”

One participant indicated that diagnostic imaging was important for her to ensure that her family physician would be able to know what the problem was and what to do:

“Well when she talked to me about what I had and all that, I wanted to know more and have a more in-depth exam to be sure of what it was and what had to be done.” (PT02, woman, long head of the biceps and RC tear).

Most were initially prescribed either X-rays, a diagnostic ultrasound or a magnetic resonance imaging. However, some participants found that imaging was used without a specific indication and used as a substitute to a clinical examination or reasoning, thus not completely meeting participants’ expectations.

A participant also mentioned that no explanations were provided after diagnostic ultrasound:

“And listen, the guy who did the ultrasound he was very… I was a bit like another number to him. He said: “complete tear”. That’s it. Complete tear of what? And he left…” (PT04, man, RC tear).

For most participants, these investigations led to treatments, such as intraarticular injections or calcific ultrasound-guided lavage, that did not result in improved outcomes, as mentioned by this participant:

“So, when she did the X-ray, they found that I had two big areas of calcific tendinitis in my shoulder, in the supraspinatus […] The calcium deposits were pretty big, I think it was something like 20 mm maybe. […] So, she sent me for a lavage. After that, it was like a nightmare. The pain really became much stronger.” (PT12, woman, RC tendinopathy/bursitis).

These quotes indicate that participants need a clear diagnosis and to know what is going on within their shoulder, and they assume that diagnostic imaging is required to understand the cause of their shoulder pain. Several participants were dissatisfied with the explanations provided or the outcome of the treatments they received after their imaging results.

### Theme 4: *Please take some time with me so that I can understand what to do!*

In addition to receiving a diagnosis and explanations about what is going on with their shoulder, most participants expected their primary care clinician to inform them about what to do to improve or solve their shoulder pain. It was also important for them that their healthcare clinician took enough time to provide these explanations.

One participant clearly indicated what her expectations were for the treatment of her shoulder pain:

“I know that for me, it’s to try to get some education to start and know how, you know, how, what I can’t do, what I can do, […] what can help not make it worse? Having exercises also, to be able to start take care of the problem itself right away…” (PT03, woman, glenohumeral instability).

This participant was satisfied because after her first meeting with a physiotherapist, she was given exercises and other treatment options and the physiotherapist scheduled a follow-up:

*“First, I would tell you, that’s what you have, at the first, at the evaluation. I left with exercises. So that, that was done straightaway. After, we added manipulations. We added some dry needling to see how it would work combined with the exercises, combined with… […] Then, I saw him the week after just to see how I was doing with the new exercises.”*

(PT03, woman, glenohumeral instability).

Two other participants were satisfied with the listening and communication skills of their primary care clinician:

“She takes the time to listen to me, and to do the physical exam also. And for example, if I say, I tell her that I want some physio then she explains to me: “Try to find a physio, [who has] some expertise, yes. Yes. I think she’s nice, my family doctor.” (PT12, woman, RC tendinopathy/bursitis).

Regarding communication skills necessary to achieve a common goal, another participant mentioned:

“So, then she was like “Do we agree on a common goal that you can sleep in the coming weeks without, you know, pain?” So, I was like “Oh yes! That was still… especially for my mood. It would be better.” So yes. So, we didn’t have the same first, overall goal with this one. But the rest was basically the same.” (PT03, woman, GH instability).

Another participant felt a high level of self-efficacy because the exercises provided by the physiotherapist allowed for the self-management of her condition:

“So, my experience was that I… there were a lot of physical exercises, also, associated with… with my problem. So, right away, I could… be… I felt in control of my improvement because I could do the exercises. […] as a patient, I will be proud to be able to tell my doctor: “Hey doctor, I took care of myself.” It’s a… it’s… it’s silly but it’s a bit like that.” (PT11, woman, RC calcific tendinitis).

Other participants were dissatisfied with the management of their shoulder condition. One participant felt that the time allowed with the physicians was too short, which made him feel like he was not important.

“So then, when I made the appointment they told me, OK so the doctor can only see you for 15 min. […] Eh wait a second, 15 min, you know it’s like when you start there, at 15 min, my input in the decisions, in the consultation with the doctor is pretty limited. I tell myself, […] am I really important or I have 15 min […] to tell my story and gogogo […].” (PT07, men, RC tendinopathy/bursitis).

Two participants also reported being disappointed and anxious because of the lack of interprofessional communication between their family physician and the physiotherapist, as stated by one participant:

“I ask myself how will they be able to fix my problem, and I am the one doing the back and forth between them. I am not supposed to, they’re in the same [building] and they can’t communicate […] I ask myself how they are going to fix my problem.” (PT07, men, RC tendinopathy/bursitis).

One participant expressed concerns about the lack of explanations provided by her family physician related to her prognosis:

“So that was when I needed information. And also to… what are my limits? How far will this thing go? Where does it stop? Will I become disabled for X years? What are the side effects also if it’s not treated?” (PT05, woman, adhesive capsulitis).

Several participants mentioned the need to know what to do to resolve their shoulder pain. The participants who were able to establish a common goal with the primary care clinician, who felt listened to and who received tools to self-manage their pain had a positive experience. Conversely, participants who had a negative experience were unsatisfied of the lack of explanations about their prognosis or treatment options or felt they did not have enough time with the physician to discuss their shoulder pain.

## Discussion

This qualitative study explored the expectations and experiences of individuals consulting in primary care for the management of shoulder pain. The themes identified were: 1) *I can’t sleep because of my shoulder*; 2) *I need to know what is happening with my shoulder;* 3) *But… we need to really see what is going on to help me!*; and 4) *Please take some time with me so I can understand what to do!*.

One key finding of the study was that participants waited until they had significant disabilities due to their shoulder pain before seeking care. They did not feel that there was anything the primary care provider could do to help them and finally decided to consult when they could not tolerate the pain anymore. This resulted in a median waiting period of eight months between the onset of shoulder pain and their first appointment in primary care. As reported in a systematic review of qualitative studies exploring the experiences of patients living with shoulder pain [19], participants were significantly distressed about their shoulder pain at the time of consultation. Frustration, sleep disturbance and difficulties carrying out activities of daily living because of shoulder pain were reported by patients from our study and many other studies included in the aforementioned systematic review [19].

Many participants did not feel confident that their family physician could help them manage their shoulder pain, which potentially resulted in a long period of time before consulting. This raises some concerns since long-lasting symptoms and severe pain are associated with a poorer prognosis [5, 6]. Other qualitative studies on the experiences of patients living with shoulder pain did not report that participants waited for a significant amount of time before consulting a care provider, or that they did not feel confident that something could be done to manage their pain [19, 27]. However, there have been previous reports that patients with various medical conditions often avoid seeking medical care because of unfavourable experiences, a low perception of the need to seek care, or because they thought that symptoms would improve over time [38–40]. Traditional barriers to healthcare access, such as high costs, lack of health insurance and time constraints, are also cited as reasons not to seek care [38]. To ensure a favourable prognosis, patients should be encouraged to consult early on, before shoulder pain becomes debilitating [5, 6]. However, potential barriers to healthcare access for shoulder pain should be studied more thoroughly as the inability to access healthcare may discourage patients from consulting early [38]. Interestingly, none of the participants in our study reported the lack of access to a family physician as a reason to wait before seeking care. However, some participants mentioned the costs of physiotherapy treatments as a barrier to seeing a physiotherapist in primary care.

A second key finding of the study was that participants expected to receive a clear diagnosis. The importance of understanding why they feel pain in their shoulder was reported by participants in another systematic review of qualitative studies exploring patients’ needs [19]. Some of our study participants said that they did receive a diagnosis. However, the explanations on the diagnosis were often considered lacking, leaving patients with the impression that they still did not clearly understand the cause of their shoulder pain. Participants from a previous study also reported that no diagnostic was provided, that the clinician demonstrated uncertainty in explaining the diagnosis or that the family physician would rely on diagnostic imaging to confirm the source of shoulder pain [27]. Moreover, many participants from our study were referred for diagnostic imaging instead of having their condition clearly explained or having a full clinical assessment. Clinicians should never use imaging tests to replace clinical examination [41]. According to a recent study, the physical examination component is often discarded by various clinicians at the time of initial evaluation [42]. Performing a clinical examination, including a history of the problem, a subjective questionnaire and an objective examination for shoulder pain, is needed to make a diagnosis and select therapeutic options according to several evidence-based guidelines [14, 43]. In our study, participants’ dissatisfaction with the diagnosis provided may have been influenced by challenges that primary care clinicians face in providing an accurate diagnosis based on their own clinical examination, such as limited consultation time as well as poor skills and confidence in assessing patients with a musculoskeletal disorder including shoulder pain [44–46].

Several participants from our study also expected to be referred for diagnostic imaging because they believed imaging tests were needed to diagnose their shoulder condition and to understand the exact cause of their pain [34]. This is in agreement with results from a systematic review reporting that according to patients living with shoulder pain, an imaging test was necessary to determine their shoulder pain diagnosis, expressing frustrations when their beliefs were challenged by clinicians [19]. However, several imaging studies suggest that structural lesions observed on diagnostic imaging are often not associated with patients’ pain complaints [47–53]. Indeed, a study evaluating the prevalence of MRI incidental anatomical findings in symptomatic compared to asymptomatic shoulders of patients presenting with unilateral shoulder pain showed no significant difference in the prevalence of lesions between both groups [53]. Full-thickness rotator cuff tears can also be detected in up to 8% of asymptomatic patients, with prevalence increasing with age [51]. In this context, diagnostic imaging for musculoskeletal disorders, including shoulder pain, should only be used to confirm a serious pathology or when diagnostic imaging results are expected to change or to tailor patient care [12]. Unnecessary diagnostic imaging induces additional delays in treatment, economic costs and leads to overdiagnosis and overtreatment [54, 55].

Therefore, diagnostic imaging for shoulder pain management should be carefully considered and results should be discussed with the patient. Issues faced by patients in understanding pain mechanisms, especially in older individuals with a lower education level, need to be considered when managing shoulder pain patients [56, 57]. Since patients’ expectations of primary care management for shoulder pain, including diagnostic imaging expectations, can affect outcomes, primary care clinicians should rely on the therapeutic relationship they have with their patients to explain the reasons why diagnostic imaging is not necessary to manage their condition [26]. Indeed, research has shown that a clinical consultation involves a negotiation process between the patient and his or her healthcare clinician, which can be facilitated by a strong therapeutic relationship [58, 59]. However, primary care clinicians may lack time to initiate the negotiation process and discuss about pain mechanisms and the implications of unnecessary diagnostic imaging with their patients [60]. Educational materials with information on the health and social consequences of imaging could be developed for patients to facilitate this discussion [61].

The last significant finding from our study is that participants were expecting to learn what to do to improve their shoulder pain and that the communication skills of the primary care provider played an important role. Several participants reported being satisfied with their primary care consultation when they felt listened to and when they were able to agree on a common, consensual care plan with their primary care clinician. Participants were less satisfied when they felt that the clinician did not take enough time with them or when they did not receive enough explanations related to the prognosis or their treatment options. These findings are in agreement with a recent study indicating that patients expect a detailed discussion on management options and prognosis and they expected to receive also reassurance in relation to their health problem [27]. Participants from our study felt satisfied when the primary care clinician offered them treatment options, including exercises. Indeed, active rehabilitation is a very important part of shoulder pain management, according to high-quality clinical practice guidelines [43]. However, qualitative studies on patients’ experience with the prescription of exercises for shoulder pain have found that some patients find these exercises challenging [19]. This was especially true for patients who believed that their pain was caused by damage in their shoulder [19]. Education on why exercises are needed and effective, the pain level to expect during exercises and how to modulate shoulder pain should be provided to the patient by the primary care clinician [12]. Primary care clinicians should take time to address patients’ prognosis and treatment options, and take time to provide reassurance [62]. This, again, can only be achieved when there is a strong therapeutic relationship between the patient and his care provider and with shared decision-making [62, 63].

### Strengths and limitations

The findings from the present study allowed us to define patients’ expectations and experiences of their first clinical consultation for shoulder pain. However, some limitations must be acknowledged. It is possible that some aspects of the primary care consultation were forgotten by participants, considering that the first consultation with the primary care clinician happened a median of 12 months before the interview. However, because of the long pain duration and time since the first consultation, we had a broader view of participants’ experiences in primary care for shoulder pain compared to participants that would have just recently consulted for their shoulder pain. Another limitation of our study is that patients knew that the interviews were performed by a physiotherapist, which may have influenced their responses, but our results are fairly consistent with other studies exploring patients’ experiences of shoulder pain management [19, 27]. Also, our interview guide was not reviewed by stakeholders. However, our guide was developed based on a previously used one which was reviewed by a clinician and a patient [34]. Our study also has major strengths. We recruited participants that consulted a clinician in primary care who had different shoulder etiologies, onset of shoulder pain and stages of recovery. The interview guide was based on one used in a previous high quality qualitative study and was adapted by our research team, which included several experienced qualitative researchers [34]. This gave an in-depth perspective of expectations and experiences of people living with shoulder pain who consulted in primary care.

### Implications for practice

An optimal management of shoulder pain according to recommendations from CPGs is crucial, especially considering that some patients consult with high levels of pain and disability present for a relatively long period. Patients with shoulder pain should be encouraged to consult earlier after the onset of pain, but the exact reasons behind these delays do require further research.

Diagnostic imaging is not recommended in the management of most shoulder pain disorders [12], but patients often expect to be referred for these tests. Since expectation can affect patients’ management and outcomes [58], interpersonal and communication skills are crucial for the clinician to engage in proper shared decision-making with the patient regarding the use of diagnostic imaging. Properly training clinicians in such interventions and offering tools to support providers and inform patients are potential solutions [63]. Moreover, a strong therapeutic relationship and more time with the patient could give primary care clinicians the opportunity to discuss the cause of shoulder pain with their patients and provide a management plan that meets evidence-based recommendations. Performing a proper clinical examination and providing a shoulder diagnosis could also reassure patients, as highlighted by a recent qualitative study [27]. Considering the current lack of entry to practice training in the management of musculoskeletal disorders for most family physicians in Canada [64], upgrading their training to better develop clinical examination skills and improve the differential diagnosis of musculoskeletal disorders should be a priority. This added training should also consider the specific context of primary care that involves a busy practice and limited clinical time with patients [65].

## Conclusion

Four themes related to patients’ expectations of shoulder pain primary care consultations were identified in our study. An unsuspected key finding is that patients waited until the pain was debilitating to consult, which may affect their prognosis. Several participants that sought a primary care consultation for their shoulder pain expected that the clinician would provide a diagnosis for their shoulder pain, but also believed that a diagnostic imaging test was necessary to explain their pain and shoulder condition. A discussion between primary care clinicians and patients may thus be necessary since diagnostic imaging is not recommended in most cases of shoulder pain. Participants expressed the need for indications on their prognosis and how to manage their shoulder pain, but several reported that these expectations were not met.

### Supplementary Information

**Additional file 1.**

## Data Availability

The datasets used and/or analyzed during the current study are available from the corresponding author on reasonable request.

## Abbreviations

RCRSP: Rotator cuff-related shoulder pain
GH: Glenohumeral
CPGs: Clinical practice guidelines
NSAIDs: Non-steroidal anti-inflammatory drugs
COREQ: Consolidated criteria for reporting qualitative research
CIUSSS: Centre intégré universitaire de santé et de services sociaux
