# Supplementary material for: “I wanted to know what was hurting so much”: a qualitative study exploring patients’ expectations and experiences with primary care management
Source: BMC Musculoskelet Disord. 2023 Sep 26;24:755. doi: 10.1186/s12891-023-06885-x (PMC10521438; doi:10.1186/s12891-023-06885-x)
Supplement: Supplementary file 1 — Additional file 1. [file 12891_2023_6885_MOESM1_ESM.docx]

**Supplementary material: Semi-structured Interview guide**

**Opening remarks**

Hello,

I wish to thank you for agreeing to take part in this research project.

My name is and I will be conducting your interview today.

**Aims of the project**

Before starting the discussion, I would like to remind you briefly about the project you are involved in.

Shoulder pain affects up to 2 out of every 3 individuals over their lifetime. This condition can become chronic and lead to disability. The overall objective of our project is to develop an intervention to improve the management of patients with shoulder pain at the first line of treatment, and to improve the collaboration between physicians and other healthcare professionals, such as physiotherapists.

To ensure that the intervention is appropriate for Quebec’s family physicians, healthcare professionals and patients we will conduct structured interviews with patients who have experienced shoulder pain, as well as discussion groups with physicians and physiotherapists.

This information will allow us to design an intervention that is appropriate for family physicians within the framework of the Quebec healthcare system.

During this interview, we would like to get a sense of your past experiences and your expectations about the management of your shoulder pain by your primary care family physician.

I will ask you various questions on your experiences and expectations about the management of your shoulder pain. There are no right or wrong answers. It is possible that I will ask for specifications as needed. Do you have any questions?

I will now begin the recording.

| **Concept** |  | **Question** | **Prompts** |
| --- | --- | --- | --- |
| Personal experience | Q1 | Can you describe your pain/shoulder problem? | How did the pain start?  What was the triggering event for your pain?  How long have you been feeling pain? |
|  | Q2 | Can you describe your pain experience/shoulder problem? | What was the impact of your pain on your daily life?   - ADL - Work - Social life - Family - Mood   What does your shoulder pain make you feel? (emotions you feel)? |
| **I will now ask you questions more specific to the first time when you went to see a *family physician* about your shoulder problem.** | | | |
| Motivations | Q3 | Can you tell me what prompted you to consult your family physician about your shoulder pain? | Who did you see first?  After the pain first started, how long did you wait before *making* an appointment?  Why?  How long was the wait time to *get* an appointment?  How many times did you see your family physician for your shoulder pain?  Did you see other physicians or healthcare professionals?  -Which ones? |
| Shared decision making | Q4 | What do you think is causing your pain? | What caused/influenced you to believe this?  Which diagnosis did you receive? From whom?  How much did your diagnosis impact the way you use your arm?  Did you expect to get a diagnosis about your shoulder pain?  How confident were you in your physician’s diagnosis? |
| Expectations | Q5 | Can you tell me about your expectations **for your first appointment** with your family physician**?** | How long did you expect the appointment to last?  What did you expect the clinical examination to be like?  What explanations and information did you expect the physician to give you? |
|  | Q6 | Can you tell me what your expectations were in terms of the care and treatment you felt you **needed**? | Did you have a preference(s) about the care you would receive?  *Did you expect a referral for medical tests or for imaging? (Why?)*  *Did you expect a referral to a specialist? (Why?)*  *Did you expect a referral to a healthcare professional? (Why?)*  *Did you expect a prescription for medication? (Why?)*  *Did you expect to receive an injection for pain (Why)?*  How open were you to these other treatment options?  What influenced your expectations about your care? |
| Shared decision making | Q7 | Can you tell me about the **treatment options** offered by your family physician? | What was the recommended sequence for these treatment options?  Were you informed on the drawbacks and benefits of each option?  Which treatment options have you already tried in the past for other problems/pain?  How did these past experiences shape your expectations?  Which of the treatments offered would you prefer?  Which treatment are you most concerned about?  How confident were you in your choice of treatment?  To what extent did you feel included in the decision-making process?  How important was it for you to be included in the decision process?  Which factors influenced your decision when choosing a treatment for your shoulder pain? |
|  | Q8 | Can you tell me about your experience with the treatments you received? | Which factors influenced your experience?  To what extent do you think you received the best possible treatment for you?  Did you feel support from your loved ones about the choice of treatment that was recommended or that you made?  How motivated were you to put into practice the advice and treatments you received?  How did your relationship with your physician impact your motivation to follow his/her recommendations and treatments for your shoulder pain?  How long did you have to wait for treatment (or for the referral)?  How did you feel about this wait time? |
| Expectations in terms of recovery | Q9 | To what extent did the care and the treatment you received meet your expectations? | How confident were you about the care and treatments recommended by your family physician?  What information did you receive about the length of your recovery?  To what extent can you use your shoulder normally now?  (*Do you still have shoulder problems*?)  Overall, was your care experience with the family physician positive or negative?  What could have improved your experience? |
